# TcellSubC: An Atlas of the Subcellular Proteome of Human T Cells

**DOI:** 10.3389/fimmu.2019.02708

**Published:** 2019-11-26

**Authors:** Rubin Narayan Joshi, Charlotte Stadler, Robert Lehmann, Janne Lehtiö, Jesper Tegnér, Angelika Schmidt, Mattias Vesterlund

**Affiliations:** ^1^Unit of Computational Medicine, Department of Medicine Solna, Center for Molecular Medicine, Karolinska Institutet, Karolinska University Hospital and Science for Life Laboratory, Stockholm, Sweden; ^2^Department of Protein Sciences, School of Engineering Sciences in Chemistry, Biotechnology and Health, KTH - Royal Institute of Technology, Science for Life Laboratory, Solna, Sweden; ^3^Biological and Environmental Sciences and Engineering Division, Computer, Electrical and Mathematical Sciences and Engineering Division, King Abdullah University of Science and Technology (KAUST), Thuwal, Saudi Arabia; ^4^Department of Oncology and Pathology, Karolinska Institutet, Science for Life Laboratory, Solna, Sweden

**Keywords:** subcellular fractionation, subcellular localization, CD4 T cells, TCR stimulation, protein translocation, mass spectrometry-based proteomics

## Abstract

We have curated an in-depth subcellular proteomic map of primary human CD4+ T cells, divided into cytosolic, nuclear and membrane fractions generated by an optimized fractionation and HiRIEF-LC-MS/MS workflow for limited amounts of primary cells. The subcellular proteome of T cells was mapped under steady state conditions, as well as upon 15 min and 1 h of T cell receptor (TCR) stimulation, respectively. We quantified the subcellular distribution of 6,572 proteins and identified a subset of 237 potentially translocating proteins, including both well-known examples and novel ones. Microscopic validation confirmed the localization of selected proteins with previously known and unknown localization, respectively. We further provide the data in an easy-to-use web platform to facilitate re-use, as the data can be relevant for basic research as well as for clinical exploitation of T cells as therapeutic targets.

## Introduction

CD4+ T cells (T helper cells) are the most abundant lymphocytes in peripheral blood and crucial modulators of the adaptive immune response. T lymphocytes are important targets for several chemo- and immunotherapeutic treatments against cancers, infections, autoimmune diseases, allergies (1, 2) and transplant rejection (3). Recent developments in “omics” technologies have opened unparalleled avenues in understanding the biology of immune cells and identifying proteins with unexplored cellular functionalities. Since the subcellular localization of a protein is vital for its function (4), determination of precise subcellular location/s can be invaluable in understanding a protein's biological function. While determination of subcellular location by tagged proteins (5) and antibody-based detection have been successful for targeted approaches (6), mass spectrometry (MS)-based proteomics methods provide high coverage to generate unbiased proteome-wide subcellular location data. For example, recent advancements in MS techniques have led to curation of high resolution maps of the subcellular proteome in human cell lines (7, 8), murine pluripotent stems cells (9), rat tissues (10) and yeast (11). Although the general classification of the subcellular proteome can be immensely improved by these studies there is a lack of context-specific classification of the subcellular proteome for primary human cells, including lymphocytes. Present efforts to classify the subcellular proteome of CD4+ T cells are mainly limited to profiling a particular subcellular fraction (12–16) or have a rather low coverage of the global proteome (17). The recent large mapping studies (7–11) have also generated several robust MS-workflows based on MS3 (9, 18–20) or extensive peptide pre-fractionation combined with MS2 (7). To enable global mapping of the subcellular location of proteins and identification of translocating proteins in response to stimulation, we generated an in-depth dataset of the spatial T cell proteome using high-resolution isoelectric focusing (HiRIEF) (21) combined with LC-MS/MS.

Here we present a proteome-wide subcellular classification of human primary CD4+ T cells into cytosolic, membrane (including organelles) and nuclear fractions in resting stage and upon 15 min and 1 h of TCR stimulation. We provide subcellular locations of more than 6,000 proteins and, to our knowledge, this serves as the highest coverage acquired for the subcellular proteome of T lymphocytes to date. Moreover, we have also profiled the proteome-wide stimulation-induced translocation, and identified 237 proteins to translocate between the subcellular fractions upon 1 h of stimulation, accomplished for the first time in primary human T cells to our knowledge.

Integrating our dataset with publicly available information on the known regulators of cellular localization like post-translational modifications (PTMs) can guide focused studies to understand the regulation of protein-shuttling in cells. We demonstrate this by integrating the translocating proteins with known stimulation induced-phosphorylations in T cells and known PTMs regulating cellular localizations, which has led to identification of several known and novel proteins and possible PTMs regulating them. Besides known TCR-induced translocations, we also identified novel ones. Furthermore, we have cross-validated selected findings of our MS study by reproducing our MS result and well-known nuclear translocation of NFATC2, as well as the novel nuclear translocation of complement component 3 (C3) upon 1 h of TCR stimulation by immunofluorescence (IF) and confocal microscopy. Altogether, we provide a resource of rapid TCR-induced subcellular proteomics in primary human T cells including validation of novel translocations that can be further exploited by the community.

## Materials and Methods

### Isolation and Stimulation of T Cells

Human peripheral blood mononuclear cells (PBMCs) were freshly isolated using Ficoll-Paque Plus (GE Healthcare) from anonymized healthy blood donor buffy coats which were purchased from Karolinska University Hospital. “Untouched” CD4+CD25– T cells were isolated from PBMCs using the CD4+ T cell Isolation Kit, human (Miltenyi Biotec) including additional depletion of CD25+ cells with CD25-specific MACS beads (8 μl per 10^7^ cells) as described earlier (22). Purity of the isolated T cells was accessed with flow cytometry and defined as CD3+ CD4+ CD25– CD8– T cells. T cells were stimulated with antibodies against CD3 (0.2 μg/ml, clone OKT3, BioLegend, LEAF grade), CD28 (2 μg/ml, clone 15E8, Miltenyi Biotec, functional grade), and goat anti-mouse Ig as a cross-linker (2 μg/ml, Southern Biotech) mimicking TCR and co-stimulation. The cells were stimulated for either 15 min or 1 h for proteomics studies or alternatively 3 h for mRNA studies. Jurkat T cells (clone E6.1) were stimulated with either above-described TCR and co-stimulation or “P/I stimulation” with Phorbol 12-myristate 13-acetate (PMA; 10 ng/ml; Sigma Aldrich) and ionomycin (375 ng/ml; Sigma Aldrich) for 1 h for imaging studies.

### Subcellular Fractionation

For each donor the isolated T cells were divided into 3 aliquots of 20 million T cells each. The aliquots were either left without any treatment (“Trest”) or TCR- stimulated for 15 min or 1 h with anti-CD3/anti-CD28 antibodies. Next, each aliquot was fractionated into cytosolic, membrane (including membranous organelles like mitochondria; and hereafter referred to as the membrane fraction) and nuclear fractions with the Qproteome Cell Compartment Kit (Qiagen). Fractionation was performed as per manufacturer's protocol except 500 μl of extraction buffer was used for the extraction of cytosolic and membrane fractions whereas 250 μl of extraction buffer was used for nuclear fraction instead of recommended volumes. Following extraction, the protein fractions were precipitated using acetone precipitation to remove contaminants from the fractionation buffers. The resulting precipitates were dissolved in buffer containing 4% SDS, 1 mM DTT, and 25 mM HEPES pH 7.6 followed by heating to 95°C for 5 min and were sonicated for 1–5 min in order to shear the genomic DNA. Protein concentrations were determined using Pierce™ BCA Protein Assay Kit (Thermo Scientific). For optimization experiments of fractionation and test of buffer compatibility with MS, aliquots of the extracts were frozen for Western Blot analysis and the remainder was frozen for MS analysis. For final proteomics studies with HiRIEF, all the material from 20 million T cells was processed for MS analysis [see section Sample Preparation for Mass Spectrometry (MS)].

### Flow Cytometry, Quantitative RT-PCR (qRT-PCR) and Western Blot

Flow cytometry, qRT-PCR and Western Blot were performed according to standard methods as described earlier (22). Western blot was performed by using antibodies against alpha-Tubulin (clone B-5-1-2, Sigma-Aldrich), GAPDH (clone 6C5, Santa Cruz Biotechnology), Caveolin-1 (N-20 polyclonal igG, Santa Cruz Biotechnology), PARP (clone C2-10, BD Pharmingen), Lamin A/C (clone mab636, ThermoFisher Scientific).

### Sample Preparation for Mass Spectrometry (MS)

Protein lysates from all the 3 subcellular fractions, 3 donors and 3 time points were digested using the FASP protocol (21, 23). The lysates were mixed with 1 mM DTT, 8M urea, 25 mM HEPES, pH 7.6 and subsequently transferred to a 10-kDa cut-off centrifugation filtering unit (Pall, Nanosep), and centrifuged at 14,000 × g for 15 min. Proteins were alkylated by 50 mM iodoacetamide (IAA) in 8 M urea, 25 mM HEPES for 10 min. The proteins were then centrifuged at 14,000 × g for 15 min followed by 2 more additions and centrifugations with 8 M urea, 25 mM HEPES. Enzymatic digestion was performed at 37°C with gentle shaking for 3 h by addition of Lys-C (enzyme: protein ratio 1:50, Wako Pure Chemical Industries) in 500 mM urea, 50 mM HEPES pH 7.6 buffer, followed by an overnight digestion with trypsin (enzyme: protein ratio 1:50, Thermo Fisher Scientific) in 50 mM HEPES, pH 7.6. The filter units were centrifuged at 14,000 × g for 15 min followed by another centrifugation with MilliQ water and the flow-through was collected. Peptide concentration was determined by Bio-Rad DCC assay and 36.1 μg of peptides from each digested fraction was labeled with TMT 10-plex reagent according to the manufacturer's protocol (Thermo Scientific; 1 TMT 10-plex per T cell donor). Additionally, 5.48 μg of peptides from each sample was aliquoted, pooled and labeled to be used as internal control for all of the 3 TMT sets. Labeled samples were pooled, cleaned by strata-X-C-cartridges (Phenomenex) and dried in a Speed-Vac. TMT labeled peptides were separated by immobilized pH gradient—isoelectric focusing (IPG-IEF) on pH 3–10 strips using the HiRIEF method (21). Peptides were extracted from the strips by a prototype liquid handling robot (GE Healthcare Bio-Sciences AB). A plastic device with 72 wells was put onto each strip and 50 μl of MilliQ water was added to each well. After 30 min incubation, the liquid was transferred to a 96 well plate and the extraction was repeated 2 more times (first with 35% acetonitrile (ACN), second with and 35% ACN, 0.1% formic acid (FA) in MilliQ water, respectively). The extracted peptides were dried in a Speed-Vac and dissolved in 3% ACN, 0.1% FA.

### MS-Based Quantitative Proteomics

Extracted peptide fractions were separated using an Ultimate 3000 RSLCnano system coupled to a Q Exactive (Thermo Fischer Scientific). Samples were trapped on an Acclaim PepMap nanotrap column (C18, 3 μm, 100 Å, 75 μm × 20 mm, Thermo Scientific), and separated on an Acclaim PepMap RSLC column (C18, 2 μm, 100Å, 75 μm × 50 cm, Thermo Scientific). Peptides were separated using a gradient of mobile phase A (5% DMSO, 0.1% FA) and B (90% ACN, 5% DMSO, 0.1% FA), ranging from 6 to 37% B in 60 min (depending on IPG-IEF fraction complexity) with a flow of 0.25 μl/min. The Q Exactive was operated in a data-dependent manner, selecting top 10 precursors for fragmentation by HCD. The survey scan was performed at 70,000 resolution from 400–1,600 m/z, with a max injection time of 100 ms and target of 1 × 10^6^ ions. For generation of HCD fragmentation spectra, a max ion injection time of 140 ms and AGC of 1 × 10^5^ were used before fragmentation at 30% normalized collision energy, 35,000 resolution. Precursors were isolated with a width of 2 m/z and put on the exclusion list for 70 s. Single and unassigned charge states were rejected from precursor selection.

### Peptide and Protein Identification

Peptide and protein identification was performed as described previously (23). Briefly, Orbitrap raw MS/MS files were converted to mzML format using msConvert from the ProteoWizard tool suite. Spectra were then searched using MSGF+ (v10072) and Percolator (v2.08), where search results from 8 subsequent fractions were grouped for Percolator target/decoy analysis. All searches were done against the human protein subset of Ensembl 75 in the Galaxy platform. MSGF+ settings included precursor mass tolerance of 10 ppm, fully-tryptic peptides, maximum peptide length of 50 amino acids and a maximum charge of 6. Fixed modifications were TMT-10plex on lysines and peptide N-termini, and carbamidomethylation on cysteine residues, a variable modification was used for oxidation on methionine residues. Quantification of TMT-10plex reporter ions was done using OpenMS project's IsobaricAnalyzer (v2.0). PSMs found at 1% FDR (false discovery rate) were used to infer gene identities.

Protein quantification by TMT 10-plex reporter ions was calculated using TMT PSM ratios to channel 131 (the internal standard) and normalized to the sample median. The median PSM TMT reporter ratio from peptides unique to a gene symbol was used for quantification. Protein false discovery rates were calculated using the picked-FDR method using gene symbols as protein groups and limited to 1% FDR.

### Identification of Translocating Proteins

To identify potential stimulation-induced translocations we analyzed the MS data using paired analysis in Limma (24) and the DeqMS R-package (25). The list of overlapping proteins from the 3 sets (3 donors, *n* = 7,122 proteins), with full quantitation in all channels (*n* = 6,572 proteins was imported into and used as a matrix. The PSM count table was generated by taking the median number of PSMs used for identification across the 3 TMT-sets. Paired analysis was carried out (within donor as a pair) and the three different time points (resting, 15 and 60 min) within each fraction being compared. When selecting the candidate translocating proteins we utilized *P*-values, not corrected for multiple testing, as a measurement of variability and fold changes as a measure of effect size, and we filtered for potential candidates by a combination of these two measures. In addition to these cutoffs, we only considered proteins that changed in two fractions in opposite direction at the same time. Specifically, we defined the potentially translocating candidate proteins as proteins which had a |log2FC|>0.201 in one direction in one fraction and a |log2FC|>0.201 in the opposite direction in another fraction, together with a DeqMS calculated *P* < 0.05 in each of the fractions (Table S1 and Figures S1, S2).

### Data Visualization and Integration

**Figure 1:**
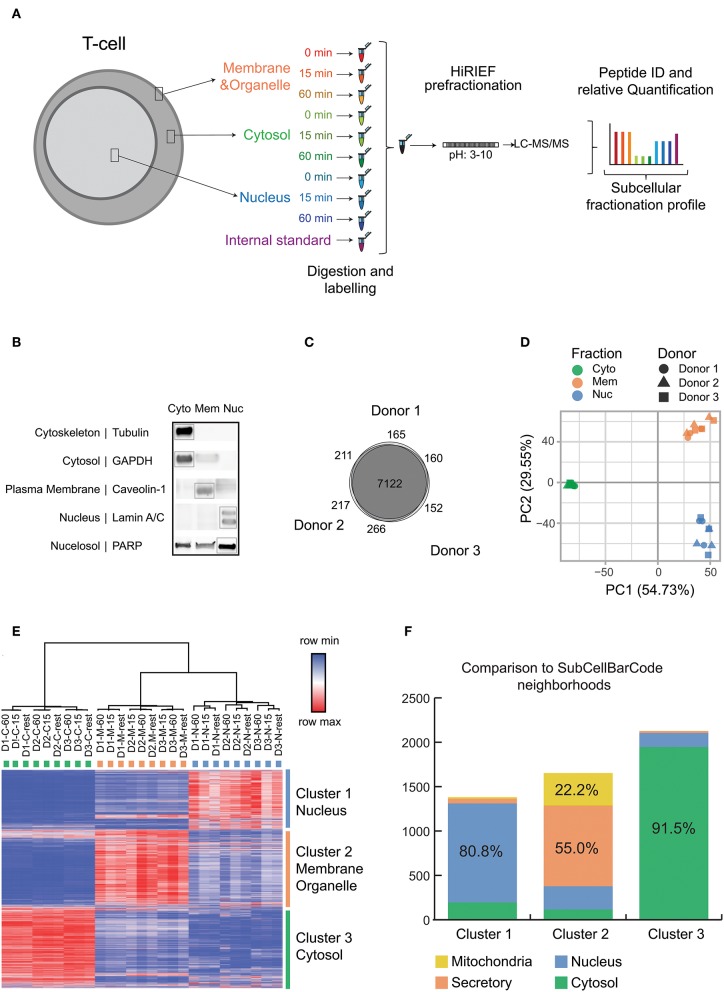
Experimental setup and quality control data for subcellular fractionation and LC-MS. **(A)** Overview of the subcellular fractionation and LC-MS workflow. CD4+ T cells were stimulated for 15 min or 1 h with cross linked anti-CD3/anti-CD28 antibodies (TCR stimulation) or processed as untreated. The cells upon fractionation were analyzed in MS as represented in the workflow. The subcellular fractions and time points of activation are represented by individual colors. The workflow was carried out individually for each donor/biological replicate (9 samples per donor) with the internal standard being the same pool of samples in all 3 runs/donors. **(B)** The figure is a representative immunoblot of the 3 subcellular components after fractionation probed with antibodies against markers of specific subcellular location as represented. **(C)** The total number of unique proteins (collapsed to gene ID) identified by at least 1 PSM for each donor and the overlap is depicted as Venn diagram. **(D)** Principle Component Analysis was performed on the TMT intensity ratios of individual components and time points from each donor normalized to the internal standard. The fractions are represented by individual colors and the donors are represented by individual shapes. **(E)** The heat map depicts log2 values of TMT intensity ratios and represented according to the indicated row normalized color scheme. The columns are clustered by average linkage method using 1 minus Pearson correlation. The rows are clustered by k means clustering (k = 3) by 1 minus Pearson correlation. The clusters are represented in individual colors. Proteins with full quantitation in all 3 donors were included (6,572 proteins). **(F)** The subcellular localization of proteins obtained are compared with localization from SubCellBarCode. Analysis is represented as stacked bar plot. The color scheme represents compartments as used in SubCellBarCode.

**Figure 2:**
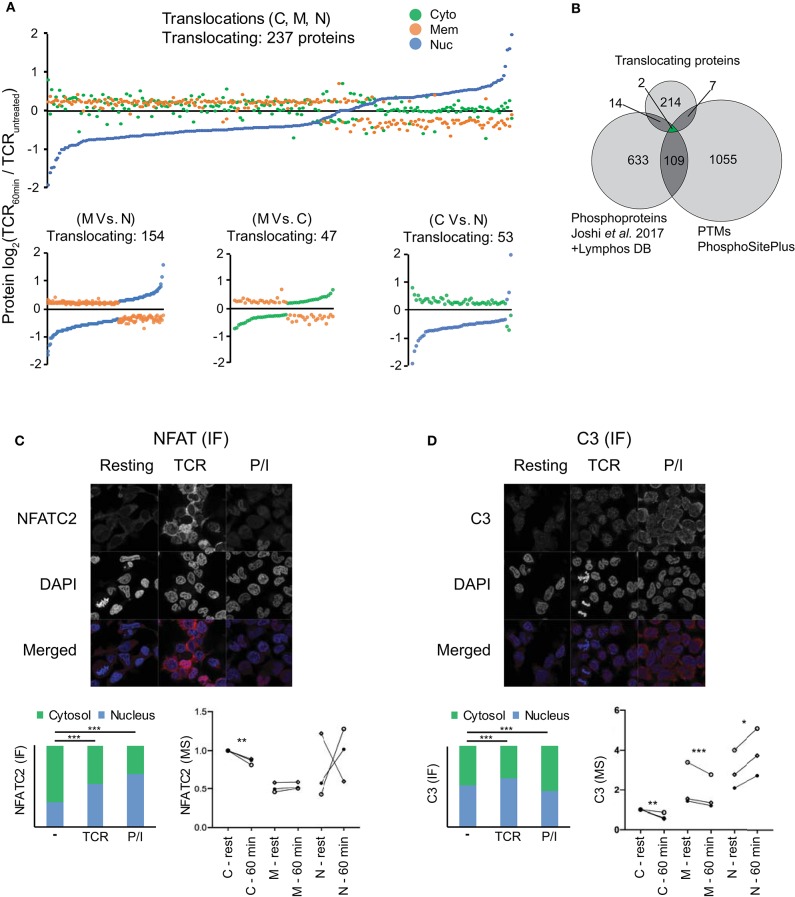
Subcellular translocation and microscopic validation of NFATC2 and C3 translocation. **(A)** Changes in the averaged log2 protein intensity in the cytosolic (C), membrane (M), and nuclear (N) compartment upon 1 h of TCR stimulation as compared to resting T cells are represented in the figure (*P* < 0.05, |log2FC|>0.201). Stimulation-induced shifts in all the 3 locations are presented in the top figure while individual comparisons are presented in each of the 3 figures on the bottom. **(B)** Venn diagram represents the overlap between the relocalized proteins, stimulation induced-phosphorylations and the PTMs regulating cellular location. Phosphoproteins were pooled from Joshi et al. (22) (changing over 25%) upon 5 min of TCR stimulation and from the LymPHOS database (changing over 50%) upon 15 min, 2 h or 4 h of PMA/Ionomycin stimulation. Additionally, PTMs experimentally reported to regulate intracellular localization from PhosphoSitePlus were used for also comparison. **(C,D)** Representative microscopic images for IF staining for NFATC2 **(C)** and complement component C3 **(D)** from Jurkat T cells in various conditions are presented along with averaged values (median) in form of stacked bar graphs (lower left) (total IF staining signal set to 1). Stacked bar graphs represent stimulation-induced redistribution of molecules between nuclear and cytoplasmic compartment, from an average of >100 cells each. *P*-values are calculated by Mann Whitney test and are indicated by stars with: ^*^P < 0.05, ^**^P < 0.01, ^***^P < 0.001. Nuclear marker DAPI in blue and target proteins in red. Additionally, MS results for subcellular relocalization upon 1 h of TCR stimulation for corresponding proteins are also presented (lower right). Donors are represented by individual symbols and the values are normalized to cytosolic protein intensity at resting stage which was further set to 1. *P* values calculated by the DeqMS R-package are indicated by stars with: ^*^p < 0.05, ^**^p < 0.01, ^****^p < 0.001.

Heat map was constructed using the web interface of Morpheus (26) using proteins classified in all the 3 locations and 3 donors (*n* = 6,572 proteins). The columns were clustered by average linkage method using 1 minus Pearson correlation. The rows were clustered by k means clustering (k = 3) by 1 minus Pearson correlation. Venn diagrams were constructed using web interface of BioVenn (27) (Figure 1C). To explore the biological and technical variation in MS result, all the proteins classified into subcellular compartment/s from 3 donors were included and convergence was plotted as a Venn diagram (Figure 2B). We performed data integration between relocalizing proteins, stimulation induced-phosphoproteins and PTMs regulating cellular localization. Proteins regulated over |log_2_FC|>0.201 in at least 2 compartments (*P* < 0.05) upon 1 h of stimulation were considered. The list of stimulation-induced phosphoproteins in lymphocytes were generated by combining phosphoproteins regulated over 25% upon 5 min of TCR stimulation (22) and over 50% upon 15 min, 2 or 4 h of P/I stimulation from the LymPHOS database (759 phosphoproteins, combined) (28). Further, PTMs which have been experimentally verified to regulate intracellular localization from PhosphoSitePlus (1174 PTMs) (29) were also considered. GO analysis was performed using the web interface of GOrilla (30). Proteins identified in all the 3 fractions and all 3 donors were used as background.

### Immunostaining, Antibodies, Image Acquisition, and Analysis

For immunofluorescence analysis, 96 well glass bottom plates (BioNordica) were coated with fibronectin (VWR). CD4+ T cell line (Jurkat) cells were seeded with 80,000 cells per well, and either stimulated with TCR or P/I stimulation or left unstimulated. After 60 min cells were fixed, permeabilized and stained as previously described (31). Primary rabbit polyclonal antibodies targeting C3 and NFATC2 (NFAT1; HPA020432 and HPA008789, Atlas Antibodies) were diluted to 2 μg/ml and detected with secondary antibody goat anti-rabbit Alexa 488 (A11034, Life Technologies) diluted to 2.5 μg/ml. A mouse monoclonal anti-alpha tubulin antibody (Ab7291, Abcam) diluted to 0.5 μg/ml and a chicken monoclonal anti-KDEL antibody (ab50601, Abcam) diluted to 2.5 μg/ml were used as common markers for the cytoskeleton and endoplasmic reticulum (ER), respectively, to allow for cell segmentation in the image analysis part. Secondary antibodies goat anti-mouse IgG Alexa-Fluor 555 (A21424) and goat anti-rat IgG Alexa-Fluor 647 (A21247) all from Thermo Fischer Scientific, were diluted to 2.5 μg/ml in blocking buffer and used for detection. Cells were counterstained with the nuclear stain DAPI at 2.28 μM solution for 10 min at RT.

The Leica SP8 confocal laser scanning microscope (CLSM), equipped with a 63x/1.4 N/A oil immersion objective, was used to acquire high resolution images of the cells to allow for evaluation of target protein distribution in resting and stimulated cells. The images were acquired at RT using the following settings: 16-bit acquisition, 2,048 × 2,048 pixels with pixel size 0.08 μm × 0.08 μm, line averaging of 2 and a pinhole of 1 airy unit (AU), scan speed 600. Laser and gain settings remained the same for all images acquired to allow for quantification and comparison of signal intensities across the cell populations. Cell Profiler (32) was used to quantify the number of cells and measure median integrated signal intensities of C3 and NFATC2 of each cell in unstimulated and stimulated cell populations. The DAPI and ER staining were used to identify cell nuclei and cell outlines, respectively, to independently measure signals within the nuclei, cytosol and entire cell. The distribution of NFATC2 and C3 between the nucleus and cytosol was compared between the cell populations to evaluate translocations upon stimulation. All intensities were normalized to the population of unstimulated cells to also allow for changes in total target expression. No image processing was done on the images. We performed statistical analysis (with *n* > 100 cells per sample) of microscopic translocations using a Mann Whitney test as described (33).

## Results and Discussion

### Subcellular Fractionation and MS Analysis

Figure 1A depicts our workflow to generate an in-depth subcellular proteomic map of primary human CD4+ T cells. T cells from 3 different donors in steady state or stimulated states (15 min or 1 h of TCR stimulation) were fractionated into 3 subcellular components, namely cytosol, membranes and small organelles and nuclei. Since human cells can be subjected to substantial donor-specific variations as compared to cell lines, we focused our analysis on the more defined subset of conventional T cells (CD4+ CD25–), hence removing recently activated and regulatory T cells by depleting CD25+ cells. Further, we evaluated the biological response of the T cells from these donors toward stimulation by studying the expression of TCR stimulation-induced *IL2* and *IFNG* mRNA upon 3 h of stimulation by qRT-PCR (Figure S3). *IL2* and *IFNG* cytokine mRNA upregulation triggered by cross-linked anti-CD3/anti-CD28 stimulation of human CD4+CD25- conventional T cells, as used here, has been previously established in our laboratory to be a suitable quality control for TCR activation at relevant time points, as assessed by calcium influx and phosphorylation of downstream signaling molecules of the NFAT, NF-kB, and AP-1 pathways (34), global increase in protein phosphorylation by phosphoproteomics (22) as well as long-term functional readouts such as secretion of IL-2, IFN-γ and other cytokines as well as T cell proliferation (34, 35). The purities of isolated protein fractions were evaluated by studying the expression of established subcellular markers by Western Blot analysis (Figure 1B). The tested protein markers were strongly enriched in the expected fractions however, it shall be noted that subcellular fractionation does not lead to perfectly pure fractions but rather an enrichment (7, 36). This may explain why some proteins can also be found in the “unexpected” fraction, such as PARP in the cytoplasm fraction; PARP appearance in the “membrane” fraction may reflect its known mitochondrial localization. Nevertheless, this method enabled reproducible fractionation from a limited amount of material from primary, small cells, in addition to buffer compatibility with MS, and this enrichment protocol was therefore used further to obtain global proteome data from subcellular fractions of T cells in resting state and upon TCR stimulation. Using high resolution fractionation and MS-based peptide detection (Figure 1A), our study identified and allocated subcellular localization for proteins corresponding to a total of 8,293 genes with a high overlap of 86% (7,122 genes) between the 3 donors (Figure 1C) which highlights the technical and biological robustness of our MS methods. More importantly both PCA as well as clustering showed clear resolution of 3 different clusters based on the 3 isolated subcellular fractions cytosol, membrane and nucleus, highlighting the reproducibility of our fractionation method across experiments and donors (Figures 1D,E). Enrichment analysis of the genes in the respective clusters according to gene ontology (GO) corresponded strongly to the genes from the expected subcellular components (data not shown). In order to compare the efficiency of our method and difference in subcellular proteomic locations in T cells we also compared the classification generated by our study with our recently published subcellular localization study; SubCellBarCode (7) which was performed on 5 different cells lines resolved into 5 different fractions. We achieved a very high overlap with the classification from SubCellBarCode between the comparable compartments (Figure 1F). This overlap was high even though here we used a different fractionation method that appeared more suitable to primary and patient-derived suspension cells with limiting starting material in order to robustly isolate basic cellular components. Further, the overlap suggests that subcellular localization of the majority of proteins in a cell is determined rather by the protein itself than by the specific cell type. In conclusion, our pipeline can present a solution in various clinical cases where input material is limited to perform detailed and robust subcellular barcoding without compromising on detection efficiency.

### Stimulation-Induced Subcellular Translocation of Proteins

In order to study the proteome-wide relocalization in T cells, we considered all the proteins that were simultaneously changing in 2 or more compartments upon 1 h of activation (*P* < 0.05) (*n* = 696). We postulated that translocating proteins should have a |log_2_FC|>0.201 in at least 2 fractions and in the opposite direction. Using this criterion, we identified 237 potentially translocating proteins (Figure 2A, Table S1). Since these proteins were simultaneously changing in reverse direction between at least 2 subcellular locations upon activation (Figure 2A; bottom) it is feasible that these proteins were relocated upon activation between the compartments. A majority of these proteins (75%) involved the membrane fraction. GO analysis suggests that these proteins are highly enriched in the components of oxidative phosphorylation like NADH dehydrogenase assembly, redox reaction and mitochondrial respiratory chain complexes. This hints at the relevance of intracellular protein shuttling in the early metabolic changes initiated upon stimulation. The translocation of STAT3 from the membrane fraction (also including organelles) to the nucleus that we observed in our study is also in line with the known oxidative phosphorylation induced-transient relocation of mitochondrial STAT3 to the nucleus (37). However, it needs to be noted that the major described role and location of STAT molecules is cytoplasmic, associated with plasma membrane-bound cytokine receptors and translocating to the nucleus upon receptor activation (37). It needs to be considered that the “bulk” nature of our isolated membrane fractions precludes more detailed statements about the exact location (within the membrane or specific organelles), yet can give indications for further targeted studies with higher resolution of membranes and organelle compartments for specific proteins of interest to the reader. Additionally, our use of cutoffs in the decision tree (Figure S1) for translocation means that some potential candidates for translocation may be disregarded due to large donor variability or small fold changes. Furthermore, since we are utilizing isobaric labeling with an MS2-based quantification with a 2 m/z isolation window, we cannot exclude the possibility that precursor mixing leads to ratio compression (38). Interfering peptide quantitative signals leading to ratio compression can be ameliorated to some extent, but not be entirely eliminated, by extensive pre-fractionation such as by HiRIEF performed by us or by pH-based reverse phase fractionation performed by others (39). Other MS studies of subcellular localization have tried to overcome similar issues by MS3 approaches (9, 18), this however comes at the cost of longer cycle times and thus fewer identifications. In both scenarios there is a risk of missing events and proteins, and with the MS2 approach we employed here we cannot exclude that some proteins are falsely classified as not translocating.

Nevertheless, our method enabled unbiased and high coverage proteomic studies from limiting primary sample material, and despite the limitations, several key elements of TCR signaling (e.g., NFKB2, NFATC1, NFATC3, STAT3, STAT5A etc.) were featured in the list of translocating proteins, in unison with the activation-induced relocations for multiple of them described in the literature (37, 40, 41). In case of the molecules identified to be relocalizing, it is known that their subcellular locations are tightly regulated by PTMs, mainly phosphorylation (37, 40, 41). To further study this point, we integrated the list of these translocating proteins with stimulation induced-phosphoproteins (in T lymphocytes) (22, 28) and with PTMs which have been experimentally verified to regulate intracellular localization (29). Twenty three proteins were identified to overlap between the studies (Figure 2B) which were enriched in signaling pathways like calcineurin-NFAT, inositol phosphate, JAK-STAT, IL-9 etc. as per GO analysis (data not shown) and well-known to be involved in TCR signaling. The detected PTMs in molecules like NFATC1, NFATC3, STAT3, STAT5A, PPP3CB, PRKCH etc. which were found to be overlapping in our study gather strong biological relevance to be studied as regulators of subcellular localization in T cells.

### Microscopic Validation of Protein Translocation on the Examples NFATC2 and Complement Component 3 (C3)

Next, we sought to verify selected proteins to translocate upon T cell stimulation by an alternative, independent method. An image-based immunofluorescence approach was used to validate results from the MS data. Firstly, we validated the technical suitability of our method and quantification approach by studying a target well-known to translocate upon TCR stimulation that is, the transcription factor NFAT (41). Several NFAT family members, as noted above, were among the list of translocating protein candidates from MS data. Although the NFAT family member NFATC2 did not pass the above thresholds set for translocating protein candidates in the MS data, we nevertheless observed the expected decrease in cytosolic NFATC2 in 3/3 donors, and a corresponding increase in the nucleus for 2/3 donors (Figure 2C). Although all donors were tested positive for activation by cytokine mRNA induction (Figure S3), it is possible that the unexpected pattern in the MS data from one of the donors may be influenced by the highly dynamic regulation of NFAT translocation. Upon TCR stimulation or triggering calcium influx with ionomycin, NFAT is dephosphorylated by the phosphatase calcineurin which leads to unmasking of its nuclear localization sequence and subsequent nuclear translocation, but different kinases confer rapid NFAT re-phosphorylation followed by nuclear re-export (34, 42). Furthermore, total levels of NFAT can change during the activation course. Nevertheless, due to availability of good antibodies and well-known nuclear translocation of cytoplasmic NFATC2 to the nucleus upon stimulation of T cells, we chose this NFAT family member for validating the technical aspects of our microscopy platform. It should also be noted that the image-based evaluation was done on the Jurkat T cell line and not primary T cells as in the MS study. This was necessary in order to accurately segment and quantify signal intensities of the cytosol and nucleus, respectively. While it is possible to segment and quantify signal in these two compartments of Jurkat cells, primary, non-blasted T cells without long-term stimulation have a very small cytosol making accurate segmentation a challenge. Hence, the validation method presented here can only capture translocations that are also occurring in an immortalized T cell line, hence are a rather general phenomenon in all CD4+ T cell subsets and independent of the already pre-activated state that an immortalized T cell line has. Furthermore, as a control, we also used P/I, a stronger stimulus that operates downstream of the TCR signaling cascades. Using this system, our imaging data also revealed higher nuclear vs. cytoplasmic signals for NFATC2 upon TCR stimulation and the nuclear fraction was even higher upon P/I stimulation (Figure 2C) (*P* < 0.001). Quantification and statistical analysis revealed that NFATC2 translocation to the nucleus vs. cytoplasm was highly significant (*P* < 0.001) in both CD3/28 and P/I stimulated cells as compared to unstimulated cells despite a general upregulation of NFATC2 especially upon TCR stimulation (Figure 2C). These data, confirming the well-known translocation of NFAT, verify the suitability of confocal microscopy for validating MS data in T cells.

After confirming the technical validity of the platform, we next aimed to validate new findings from our MS study. We focused on the complement component C3, which showed TCR stimulation-induced cytoplasm to nuclear translocation in the MS study (Figure 2D). In contrast to NFATC2, stimulation-induced translocation of C3 is not well-studied. Classically complement is viewed as serum operative component of innate immunity with extracellular activation as the major mechanism of C3 activation. However, multiple studies suggest that intracellular activation of C3 contributes to several aspect of T cell biology mainly proliferation and cytokine secretion (43–45). Although extended TCR stimulation has been suggested to induce the translocation of C3 from endosomal and lysosomal stores in T cells to the membrane and subsequent regulation of T cell activation (44), not much knowledge exists on the effect of short-term TCR stimulation on C3. Our MS data indicates that 1 h of TCR stimulation induces the nuclear translocation of C3. It is possible that this is an intermediate step prior to the shuttling of C3 to the cell surface to amplify T cell activation. Interestingly, complement receptor signaling to T cells has been observed to have crucial roles in T cell differentiation, and intracellular storage of C3 in T cells followed by transfer to the cell surface might augment such activation (46–49). Confirming the MS data, T cells had intracellular levels of C3, and confirming the translocation observed in the MS data, the quantitative data from the images of C3 staining showed slightly higher C3 abundance in the nucleus compared to the cytosol upon TCR stimulation which was clearly significant (*P* < 0.001) (Figure 2D). Interestingly, for cells stimulated with the stronger stimulant P/I, the overall expression of C3 was highly increased, although with a different subcellular distribution (Figure 2D). Overall, the imaging data for C3 obtained in Jurkat T cells concur with the MS proteomics experiments and indicate novel, cytoplasm to nuclear translocations and increase of C3 in short-term TCR-stimulated T cells.

## Perspective and Data Usage

We present a robust workflow of subcellular fractionation and high-resolution LC/MS analysis suitable for primary cells with limited availability, which may be further adapted to perform high resolution proteomic analysis in clinical settings and for rare cell types. Further, our ‘TcellSubC’ subcellular classification of 7,122 proteins can provide guidelines to follow up potential novel biological functions of the identified and translocating proteins in T lymphocytes. Besides the high-resolution proteomic mapping that “TcellSubC” provides, we also report the first TCR stimulation induced proteome wide-relocalization study in T cells with comprehensive coverage. Our results also pave the way for further experimental follow ups on studying PTMs and other mechanisms as regulators of subcellular translocation of proteins. Our database will particularly support functional studies of the novel molecules identified from several global omics and prediction studies which are getting more and more common with the advent of high throughput technologies. The subcellular location from our study can be readily used for hypothesis generation for T cell specific cellular function and can also save researchers from having to experimentally determine the subcellular location of their protein/s of interest, although experimental validation by independent methods should always be performed. As discussed above, our use of cutoffs and the MS2 approach could lead to some events or translocations being discarded or ignored in the current workflow. To enable convenient retrieval and re-analysis of the data, we have provided an easily editable document with final calculations and statistical analyses (Table S1) as well as an interactive web-platform (https://tcellatlas.kaust.edu.sa/). The mass spectrometry dataset including raw spectral files is also available for download from ProteomeXchange (PXD013284).

## Data Availability Statement

The mass spectrometry proteomics data have been deposited to the ProteomeXchange Consortium via the PRIDE (50) partner repository with the dataset identifier PXD013284, https://www.ebi.ac.uk/pride/archive/projects/PXD013284. Data are provided in a web platform for visualization and re-use at https://tcellatlas.kaust.edu.sa/.

## Ethics Statement

Human peripheral blood mononuclear cells (PBMCs) were freshly isolated from anonymized healthy donor buffy coats purchased from the Karolinska University Hospital (Karolinska Universitetssjukhuset, Huddinge), Sweden. Research was performed according to the national Swedish ethical regulations (ethical review act, SFS number 2003:460). Ethical permit for the experiments was obtained from the Regional Ethical Review Board in Stockholm (Regionala etikprövningsnämnden i Stockholm), Sweden (approval number: 2013/1458-31/1).

## Author Contributions

RJ, AS, and JL conceived the study. RJ performed all the T cell experiments, method optimisation, and subcellular fractionation. RJ performed the MS sample preparation with suggestions from MV. MV performed the bioinformatics analysis related to generation of differential ratios of the TMT labels. RJ performed the further downstream analysis with suggestions from MV, JT, AS, and JL. CS designed and performed the imaging experiments and imaging data analysis. JT, AS, and JL supervised research. RL implemented the web platform. RJ wrote the manuscript with suggestions from MV, AS, JL, and CS. All authors approved the final version of the manuscript.

### Conflict of Interest

The authors declare that the research was conducted in the absence of any commercial or financial relationships that could be construed as a potential conflict of interest.
