# Supplementary material for: TcellSubC: An Atlas of the Subcellular Proteome of Human T Cells
Source: Front Immunol. 2019 Nov 26;10:2708. doi: 10.3389/fimmu.2019.02708 (PMC6902019; doi:10.3389/fimmu.2019.02708)
Supplement: Supplementary file 2 [file Image_1.pdf]

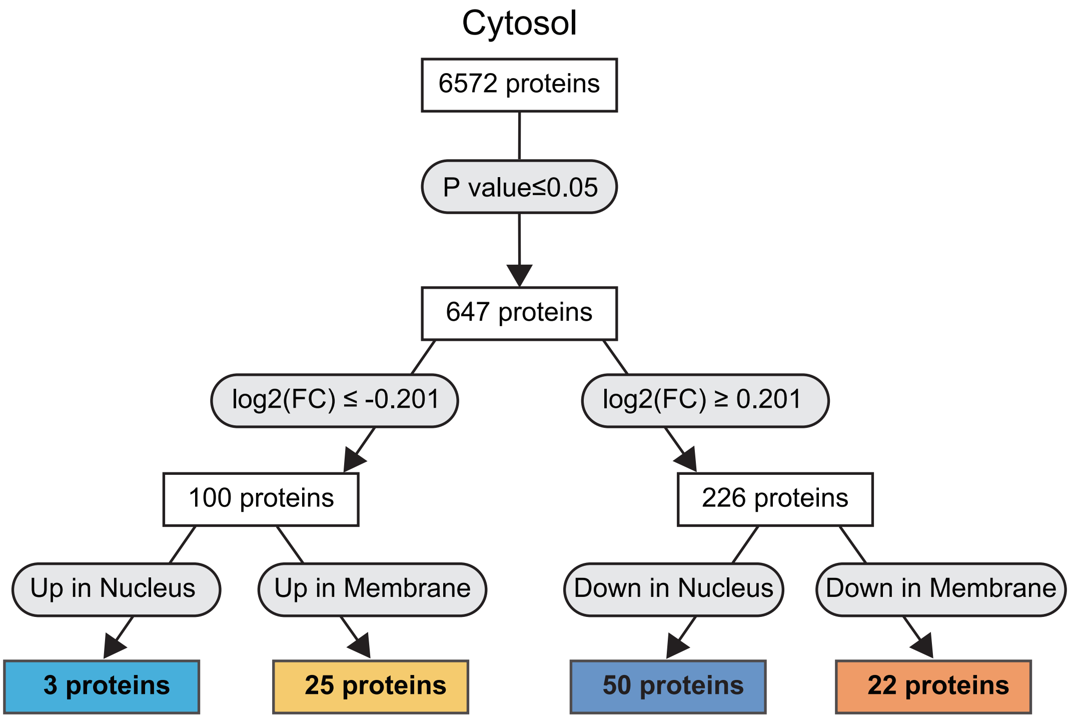

Figure S1. Decision tree structure employed to identify translocating proteins.
The decision tree employed for identification of proteins translocating to or from cytosol, as an example, is depicted. Identical filtering steps were employed for the Nuclear and Membrane compartments respectively (not depicted). Rounded, grey boxes denote filtering steps and rectangular boxes denote the number of proteins remaining after specific filtering steps. For the last round of filtering proteins found to be increased or decreased in either of the other two compartments (as compared to the cytosol and in the opposite direction) were called as translocating proteins.
