# Supplementary material for: TcellSubC: An Atlas of the Subcellular Proteome of Human T Cells
Source: Front Immunol. 2019 Nov 26;10:2708. doi: 10.3389/fimmu.2019.02708 (PMC6902019; doi:10.3389/fimmu.2019.02708)
Supplement: Supplementary file 3 [file Image_2.pdf]

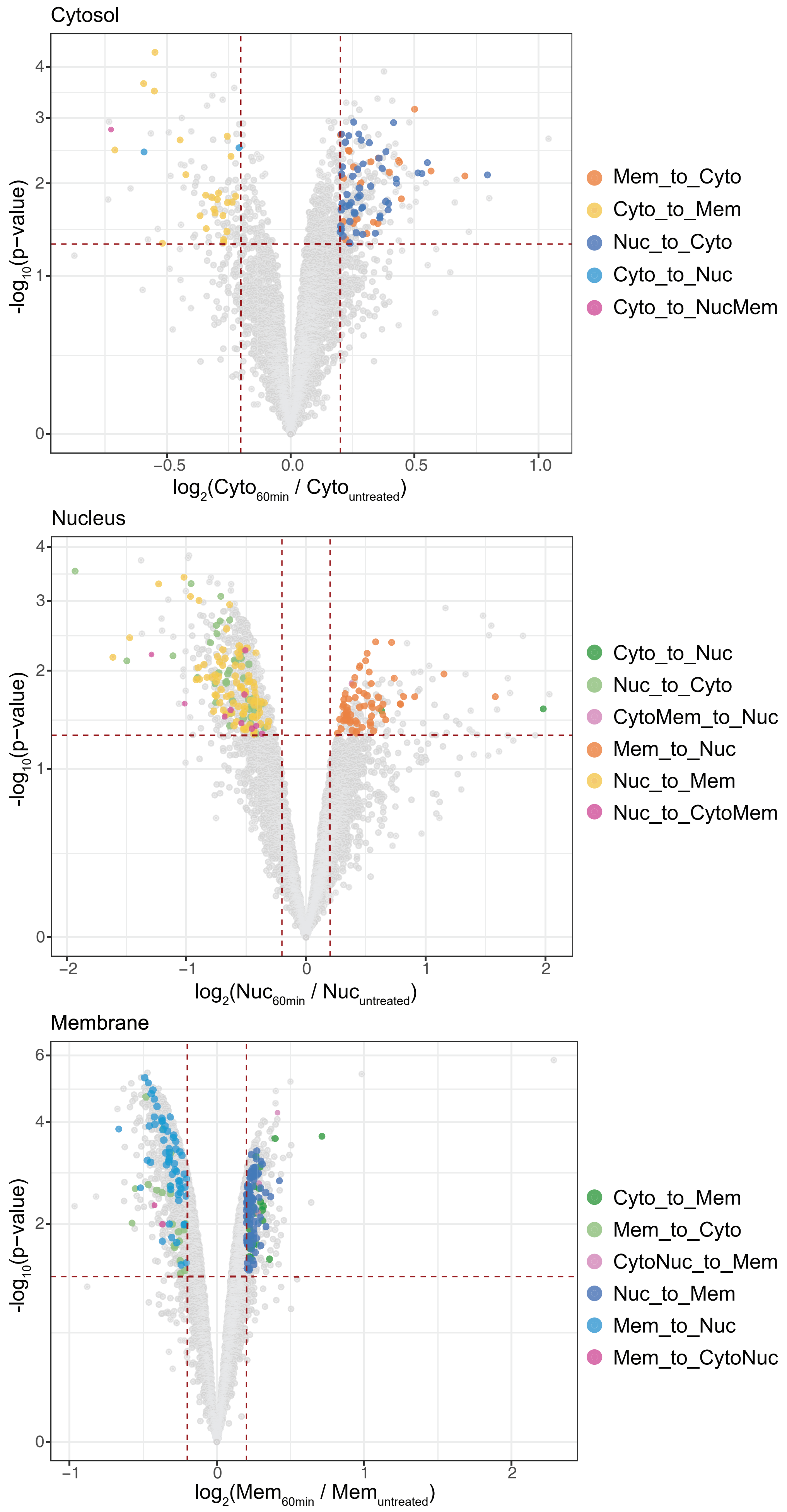

Figure S2. Volcano plots of TCR stimulated cells compared to unstimulated cells.
The log2 foldchange (60 min/untreated) for individual proteins is plotted against the DeqMS calculated p-value for each individual compartment (Cytosol, Nucleus and Membrane). Proteins that fulfilled the criteria for translocation (opposite direction of change in at least 2 compartments with an absolute log2 foldchange >0.201 as well as a p-value <0.05 in both) are highlighted and color coded based on the translocation as denoted above.
