# Supplementary material for: TcellSubC: An Atlas of the Subcellular Proteome of Human T Cells
Source: Front Immunol. 2019 Nov 26;10:2708. doi: 10.3389/fimmu.2019.02708 (PMC6902019; doi:10.3389/fimmu.2019.02708)
Supplement: Supplementary file 4 [file Image_3.pdf]

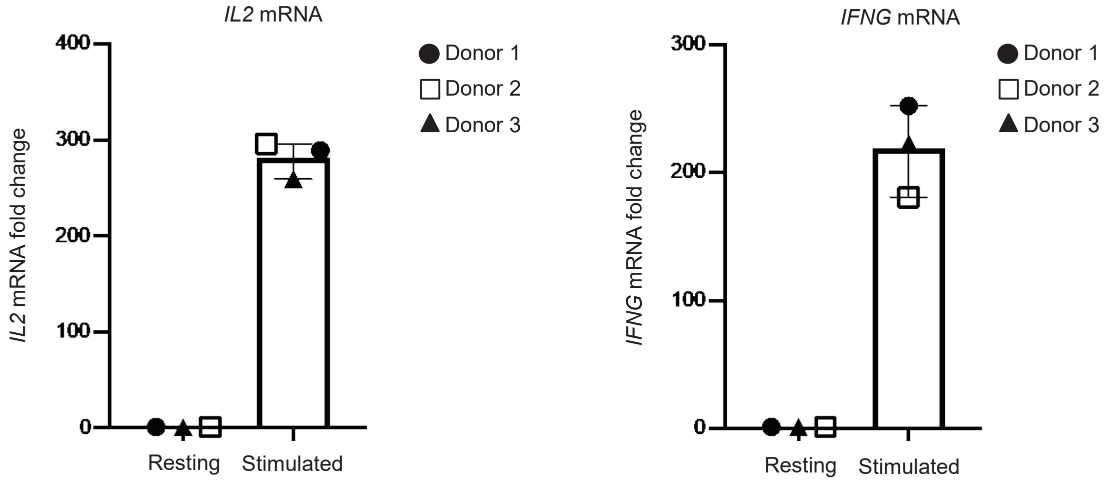

Figure S3. Quality control analysis of T cells used for proteomics
Aliquots of T cells from all the 3 donors were stimulated for 3 hours with cross linked anti-CD3/anti-CD28 antibodies (TCR stimulation) or processed as untreated prior to subcellular fractionation. The expression of *IL2* (left) and *IFNG* (right) mRNA were measured by qRT-PCR and normalized to *GAPDH* mRNA. Results are presented as fold change compared to unstimulated samples (set to 1). Donors are represented with individual shapes (mean ± range of donors).
